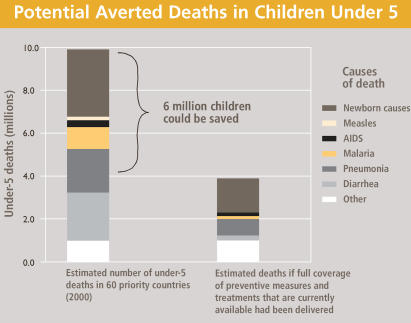# Environmental Disease: Saving the Lives of Children Under 5

**DOI:** 10.1289/ehp.115-a490

**Published:** 2007-10

**Authors:** Adrian Burton

Save the Children has compiled the first ever league table ranking 60 developing nations in their efforts to preserve the lives of their youngest citizens. This information, published in the May 2007 report *State of the World’s Mothers 2007: Saving the Lives of Children Under 5*, illustrates how even the poorest countries can implement effective solutions to prevent untimely child deaths.

The table comprises UNICEF data for all countries with at least 50,000 child deaths per year or a rate of 90 deaths per 1,000 live births in 2004, and reflects changes in under-5 mortality since 1990. The 60 countries included were home to 75% of the world’s children under age 5 years in that year, and were where 94% of all under-5 deaths occurred.

Some countries have made remarkable progress in reducing the number of under-5 deaths. Egypt, for example, achieved a massive 68% reduction, Indonesia 60%, and Bangladesh, Nepal, and the Philippines each around 50%. Thirty-five other countries also have seen improvements. However, 20 countries, including 18 in sub-Saharan Africa, have either seen no improvement or suffered increases. Iraq saw the worst decline in the world—under-5 mortality increased by 150% as a result of the deprivations imposed by civil unrest, sanctions, and war.

“The figures show much progress has been made [in many countries], but there is still much to do,” explains David Oot, associate vice president of health at Save the Children. Oot says that if certain simple, low-cost solutions were made available to all children—ensuring that a skilled person attended all deliveries, exclusive breastfeeding for the first 6 months of life, immunization against measles, use of oral rehydration solution for diarrhea sufferers, and low-cost treatment of common infections such as pneumonia and malaria—more than 6 million more lives could be saved every year. These solutions improve survival by targeting the major causes of child mortality.

He highlights that success is possible even under difficult circumstances: “Several countries with combinations of weak health services, political unrest, and low incomes have made significant progress. Nepal, for example, clearly shows what can be achieved when the political will is there and basic, cost-effective interventions are implemented at scale.” Despite an average per-capita income of just US$1,530 and years of civil unrest, Nepal has increased child vaccination coverage from 43% to 83%, has trained community health workers and parents to treat pneumonia and diarrhea at home, is now training the same community health workers to administer antibiotics to treat sepsis in newborns, and is educating women on contraception to reduce unwanted and high-risk pregnancies.

The report breaks down the rank listing for each country into the percentages of the under-5 population presently covered by the above low-cost solutions—data that could provide NGOs and government authorities with a way to focus their actions where they can be most beneficial.

However, says William Moss, an associate professor of epidemiology at the Johns Hopkins Bloomberg School of Public Health, although the five recommended solutions are essential to improving child survival, they can not prevent all child deaths. Moss says, “These strategies are drawn from a larger list of cost-effective preventive and treatment interventions that must be delivered as an integrated package”

A spokesman for Oxfam UK also noted that some countries may be unable to absorb all the available aid centered on these solutions, for example due to a lack of civil servants to oversee its distribution. Further, some countries face special threats, such as HIV/AIDS, against which these solutions would make little impact.

Although the list provides a starting point for understanding why some countries have been successful in reducing child mortality, Moss cautions that alone it may not provide sufficient information to fully guide future decision making. “A more detailed analysis might identify interventions, programs, and health system characteristics in those countries that substantially reduced child mortality, which could guide child survival strategies in countries with similar epidemiological profiles,” he concludes.

## Figures and Tables

**Figure:**
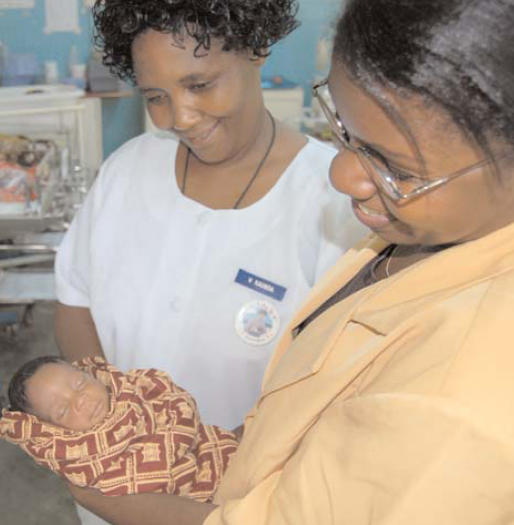
Skilled assistance at birth increases both infant and mother survival.

**Figure:**